# Investigating the Effects of Anodal Transcranial Pulsed Current Stimulation at Low Frequencies (0.5 to 5 Hz) on Corticospinal and Corticocortical Excitability

**DOI:** 10.1111/psyp.70092

**Published:** 2025-06-16

**Authors:** Mona Malekahmad, Ashlyn Frazer, Maryam Zoghi, Shapour Jaberzadeh

**Affiliations:** ^1^ Monash Neuromodulation Research Unit (MNRU), Department of Physiotherapy Monash University Melbourne Victoria Australia; ^2^ Discipline of Physiotherapy Federation University Ballarat Victoria Australia

**Keywords:** corticocortical excitability, corticospinal excitability, tPCS, low frequencies, transcranial magnetic stimulation, transcranial pulsed current stimulation

## Abstract

The mechanism underlying transcranial pulsed current stimulation (tPCS) as a non‐invasive neuromodulation technique has garnered considerable attention in recent years. However, the effects of anodal tPCS (a‐tPCS) at low frequencies remain unexplored. This study aimed to investigate the a‐tPCS effects at 0.5, 1, 3, and 5 Hz on cortical outcomes and its adverse side effects. This double‐blinded, randomized, counterbalanced crossover trial included 18 healthy young participants who completed five experimental sessions with 2 mA of a‐tPCS for 20 min in a randomized order of frequencies (0.5, 1, 3, and 5 Hz). Single‐pulse and paired‐pulse transcranial magnetic stimulation (TMS) on the left primary motor cortex (M1) was delivered before and immediately after the stimulation at rest. Sessions were spaced at least 48 h apart to minimize carry‐over effects. The results indicated that a single session of a‐tPCS at frequencies of 1, 3, and 5 Hz significantly (*p* < 0.05) enhanced corticospinal excitability (CSE), while 0.5 Hz decreased CSE compared to sham stimulation. The CSE changes at 1 and 5 Hz were associated with increased intracortical facilitation (ICF), with reduced adverse effects observed at higher frequencies. In contrast, the cortical effects of 0.5 Hz were linked to increased short intracortical inhibition (SICI) with minimal side effects. However, all frequencies except 0.5 Hz were associated with phosphenes or flashing lights during stimulation. Given the similar effects of a‐tPCS with other pulsatile current stimulation, it is plausible that tPCS could serve as a complementary or superior alternative to TMS, particularly for at‐risk and diverse patient populations. However, this claim needs further comparative studies before suggesting clinical superiority for epilepsy and neuro‐rehabilitation. Furthermore, like other neuromodulation techniques, tPCS shows potential as an affordable, home‐based treatment option. Further research is needed to establish the efficacy of tPCS relative to TMS methodologies through rigorous experimental testing.

## Abstract 2

Transcranial pulsed current stimulation (tPCS) is a non‐invasive method for modulating cortical excitability via entrainment and polarization. Our findings show that anodal tPCS below 1 Hz reduces CE through GABAergic activity, while above 1 Hz enhances it via glutamatergic mechanisms. These effects align with transcranial magnetic stimulation (TMS) but with fewer adverse effects at delta/theta frequencies. Given its safety and affordability, tPCS holds promise as a neuromodulatory intervention for conditions with altered CE.

**Abbreviations**
A‐tPCS: Anodal transcranial pulsed current stimulation
CCE: Corticocortical excitability
CE: Cortical excitability
CSE: Corticospinal excitability
C‐tPCS: Cathodal transcranial pulsed current stimulation
EEG: Electroencephalography
ES: Electrode size
EMG: Electromyography
FDI: First dorsal interosseous
GABA: Gamma‐aminobutyric acid
ICF: Intracortical facilitation
IPI: Inter‐pulse interval
ISI: Inter‐stimulus interval
LICI: Long‐interval intracortical inhibition
M1: Primary motor cortex
MEP: Motor evoked potential
MT: Motor threshold
NDCC: Net direct current component
NIBS: Non‐invasive brain stimulation
NMDA: N‐methyl‐D‐aspartate
NP: Number of pulses
PD: Pulse duration
PEST: Parameter estimation sequential testing
RMT: Resting motor threshold
SICI: Short‐interval intracortical inhibition
SI: Stimulation intensity
TC: Total charge
tACS: Transcranial alternating current stimulation
tDCS: Transcranial direct current stimulation
TMS: Transcranial magnetic stimulation
tPCS: Transcranial pulsed current stimulation

## 1 Introduction

Transcranial pulsed current stimulation (tPCS) is a neuromodulatory technique that delivers unidirectional monophasic positive rectangular pulses at varying frequencies. Unlike transcranial direct current stimulation (tDCS), which applies a continuous direct current, tPCS delivers discrete pulses separated by a predetermined pulse duration (PD) and inter‐pulse interval (IPI) (Jaberzadeh et al. 2014). The net direct current component (NDCC), determined by the pulse amplitude, PD, and IPI, contributes to static polarization by generating a sustained electric field that modulates the resting membrane potential and facilitates charge accumulation in neural tissue. Notably, tPCS with higher NDCC can induce static polarization effects by altering the resting membrane potential, similar to tDCS (Jaberzadeh et al. 2015).

Additionally, the pulsatile nature of tPCS, characterized by its on/off pattern, can induce dynamic effects that depend on frequency, resembling the underlying mechanisms of transcranial alternating current stimulation (tACS) (Malekahmad et al. 2024). Since tACS entrains neural oscillations by applying continuous, periodic polarization in synchrony with endogenous rhythms, it has been hypothesized that tPCS may similarly modulate neural oscillatory synchronization in a frequency‐dependent manner (Vasquez et al. 2016; Thibaut et al. 2017). Consequently, tPCS may influence cortical excitability (CE) and neuroplasticity through both polarization and oscillatory modulation, depending on stimulation frequency and amplitude (Malekahmad et al. 2024).

In this regard, a study reported that anodal tPCS (a‐tPCS) at 4 Hz and 75 Hz can enhance corticospinal excitability (CSE), potentially mediated by synaptic plasticity through mechanisms of long‐term potentiation (LTP). Notably, stimulation at 75 Hz with an intensity of 1.5 mA, applied to the left primary motor cortex (M1), induced significantly greater CSE changes than 4 Hz stimulation under similar conditions (Dissanayaka et al. 2022). Another study found that low‐frequency a‐tPCS at 0.5 Hz applied to M1 reduced CSE and modulated intrinsic neural fluctuations, potentially through long‐term depression (LTD) (Luu et al. 2016). Consequently, tPCS may induce excitatory effects at higher frequencies (> 4 Hz) while exerting inhibitory effects at lower frequencies of 0.5 Hz.

Neuromodulation at different frequencies of tPCS has been associated with various neurophysiological effects, including synaptic plasticity and the regulation of excitatory‐inhibitory balance (Malekahmad et al. 2024). A key mechanism underlying these effects is the modulation of N‐methyl‐D‐aspartate (NMDA) receptor activity, which contributes to synaptic strengthening and neuroplasticity (Dissanayaka et al. 2022). These processes are well documented in the context of frequency‐dependent transcranial magnetic stimulation (TMS), where NMDA receptor‐mediated calcium ion (Ca^2+^) influx plays a central role in LTP induced by high‐frequency TMS (≥ 5 Hz) (Ziemann et al. 2008; Müller‐Dahlhaus and Ziemann 2015). Consequently, high‐frequency TMS is widely used in clinical applications such as depression treatment and stroke rehabilitation (Wassermann and Lisanby 2001; Afifi 2024). In contrast, low‐frequency TMS (≤ 1 Hz) is primarily associated with LTD, which involves glutamate‐mediated pathways and is modulated by inhibitory gamma‐aminobutyric acid (GABAergic) mechanisms (Tan et al. 2018; Zhang et al. 2022). This form of neuromodulation is particularly effective for conditions characterized by cortical hyper‐excitability, such as epilepsy and tinnitus (Wassermann and Lisanby 2001). Given the NMDA receptor's known sensitivity to low‐frequency oscillations, similar mechanisms may underlie the effects of tPCS at 0.5 to 5 Hz frequencies. Understanding this relationship is essential for elucidating the broader neuromodulatory impact of frequency‐dependent stimulation and its potential therapeutic applications.

However, unlike TMS, which induces direct neuronal depolarization with frequency‐dependent effects on excitability, tPCS modulates CE indirectly by altering membrane potential or entraining oscillatory activity, similar to other tES modalities. Additionally, tPCS shares features with tACS, which has dynamic effects on neural oscillations, and tDCS, which induces more static changes in CE. While preliminary findings suggest that tPCS may induce neuroplasticity effects comparable to TMS, further empirical evidence is required to establish this relationship (Malekahmad et al. 2024). Unlike TMS, which activates neurons using suprathreshold magnetic pulses, tPCS modulates the resting membrane potentials of neurons through subthreshold electrical stimulation. Although both techniques are associated with side effects, such as the rare risk of seizure with TMS (Rossi et al. 2009; Yoshida et al. 2019; Rossi et al. 2021) and transient discomfort or skin irritation with tPCS (Jaberzadeh et al. 2014, 2015), the subthreshold nature of tPCS may confer a more favorable safety and tolerability profile, particularly for use in vulnerable populations.

As previous studies have highlighted a gap in understanding the underlying mechanisms of tPCS (Malekahmad et al. 2024), this study aimed to investigate the effects of low frequencies (0.5, 1, 3, 5 Hz, and sham) a‐tPCS on CSE and corticocortical excitability (CCE). The selection of these a‐tPCS frequencies was guided by previous findings on non‐invasive brain stimulation (NIBS). TMS, as a frequency‐dependent NIBS technique, has been shown to induce distinct neurophysiological outcomes based on stimulation frequency, eliciting LTD‐like inhibitory effects at below 1 Hz frequencies (≤ 1 Hz) and LTP‐like facilitatory effects at above 5 Hz frequencies (≥ 5 Hz) (Ziemann et al. 2008; Tan et al. 2018). Although TMS operates using suprathreshold intensities and extremely brief pulse durations, the present study sought to investigate whether analogous frequency‐dependent neuromodulatory effects could be observed with tPCS. Notably, there is a paucity of research systematically exploring the effects of tPCS within lower‐frequency ranges, particularly in 0.5 to 5 Hz frequencies. These specific frequencies were selected based on their physiological and clinical relevance that categorized into the delta band (0.5–4 Hz) and theta (4–8 Hz), which reflect commonly used electroencephalography (EEG) brainwave classifications (Kumar and Bhuvaneswari 2012; Le Bon et al. 2012). These frequency oscillations play critical roles in synaptic plasticity, neural communication, and cognitive processes, and abnormalities in these rhythms have been implicated in various neurological and psychiatric conditions. For example, atypical frequency activities have been reported in individuals with developmental attachment disorders (Carbone et al. 2024) which are associated with emotional dysregulation, depression, and anxiety (Larsen et al. 2024), as well as chronic pain and fibromyalgia (Katzman and DEL Fabbro 2024). Given that 0.5 to 5 Hz frequency oscillations are implicated not only in emotion and cognition but also in motor control and synaptic plasticity (Larsen et al. 2024), the present study seeks to address this gap by examining whether a‐tPCS induces frequency‐dependent modulation of cortical excitability within the motor cortex.

On the other hand, prior research on NIBS has typically focused on high‐frequency stimulation (e.g., gamma, beta) or static neuromodulation (e.g., tDCS), the potential for low‐frequency (0.5 to 5 Hz) a‐tPCS to modulate CE remains largely unexplored. Understanding the effects of frequency‐dependent a‐tPCS modulation on CE could provide valuable insights into its neuromodulatory mechanisms and potential clinical applications.

Therefore, the current study is structured to achieve three main objectives: (1) to investigate the effects of low‐frequency a‐tPCS within low frequencies (0.5, 1, 3, and 5 Hz) on CSE using single‐pulse TMS motor evoked potentials (MEPs), (2) to examine the potential facilitatory and inhibitory mechanisms underlying CSE changes by assessing alterations in glutamate and GABA levels, measured using MEPs induced by paired‐pulse TMS, and (3) to evaluate the potential side effects of a‐tPCS both during stimulation (online effects) and immediately post‐stimulation (offline effects) within the 0.5 to 5 Hz frequency ranges.

This study hypothesizes to clarify how different frequencies of a‐tPCS affect CS, based on the hypothesis that, like TMS, below 1 Hz (≤ 1 Hz) will produce inhibitory effects. In comparison, frequencies above 1 Hz (≥ 1 Hz) will be excitatory. These effects are expected to reflect underlying mechanisms of synaptic plasticity, specifically LTP‐like changes mediated by glutamate at excitatory frequencies and LTD‐like changes associated with GABA at inhibitory frequencies. Furthermore, we anticipate that a‐tPCS will be well‐tolerated across all tested frequencies with minimal adverse effects. By systematically comparing frequency‐specific effects, this study seeks to provide foundational insights into the mechanisms of tPCS and its potential clinical applications relative to established neuromodulation techniques like TMS. Given its affordability, ease of use, and suitability for home‐based applications, tPCS holds promise as an accessible neuromodulation option for vulnerable populations.

## 2 Material and Methods

### 2.1 Participants

Eighteen healthy participants (6 female, 12 male) with a mean age of 18–45 years (25.38 ± 6.93) volunteered and completed the Adult Safety Screening Questionnaire to confirm eligibility for TMS (Keel et al. 2001). None reported neurological conditions, neurosurgery, seizures, metallic implants, or medication use. A priori power analysis was conducted using G*Power (v. 3.1.9.7) (Faul et al. 2007) to determine the minimum sample size required to detect significant within‐subject effects across stimulation frequencies. Effect sizes were estimated from pilot data examining changes in motor evoked potential (MEP) amplitude following each frequency of a‐tPCS. Based on these estimates, the minimum required sample size to achieve 80% power at *α* = 0.05 was 16 (Cohen 2013). To ensure adequate power across all conditions and account for variability, we recruited 18 healthy participants, each of whom received all five stimulation conditions (sham, 0.5, 1, 3, and 5 Hz) in a within‐subject crossover design.

#### 2.1.1 Inclusion and Exclusion Criteria

All participants met the inclusion criteria required to be right‐handed, as assessed by the Edinburgh Handedness Inventory (https://www.brainmapping.org/shared/Edinburgh.php#) (Chipchase et al. 2012). They also had no prior experience with transcranial stimulation within 48 h preceding the sessions and abstained from caffeine, alcohol, and cigarettes on experiment days. Additionally, female participants did not undergo sessions during their menstrual cycles. This exclusion was based on evidence indicating that fluctuations in estrogen and progesterone levels during the menstrual cycle can alter CE and neuroplasticity (Smith et al. 1999), potentially confounding the effects of tPCS. Additional exclusion criteria included metallic implants in the head, indwelling body stimulators, a history of seizures, current pregnancy, and inability to complete all sessions or tolerate stimulation, as assessed through a side‐effect questionnaire during tPCS.

#### 2.1.2 Randomization and Blinding

Participant blinding was ensured by assigning numerical codes to each experimental session, with session orders determined using an online random sequence generator (https://www.random.org/) (Boutron et al. 2007). A notable challenge was the induction of phosphenes (perceived light‐flashing sensations), a common side effect of low‐frequency tPCS (Jaberzadeh et al. 2014, 2015). To address this, a frequency‐adaptable flashing light was positioned behind participants during sham stimulation to replicate the flashing effect (Dissanayaka et al. 2020). Blinding integrity was assessed by asking participants at the end of the tPCS whether they believed they had received active stimulation or sham. The responses were analyzed using a chi‐square test to determine whether participants' guesses were at chance level. A non‐significant result would indicate successful blinding. Furthermore, the researcher who conducted the statistical analysis remained blinded to participant identities and stimulation conditions by using anonymously coded data throughout the analysis.

### 2.2 Study Design

**FIGURE 1:**
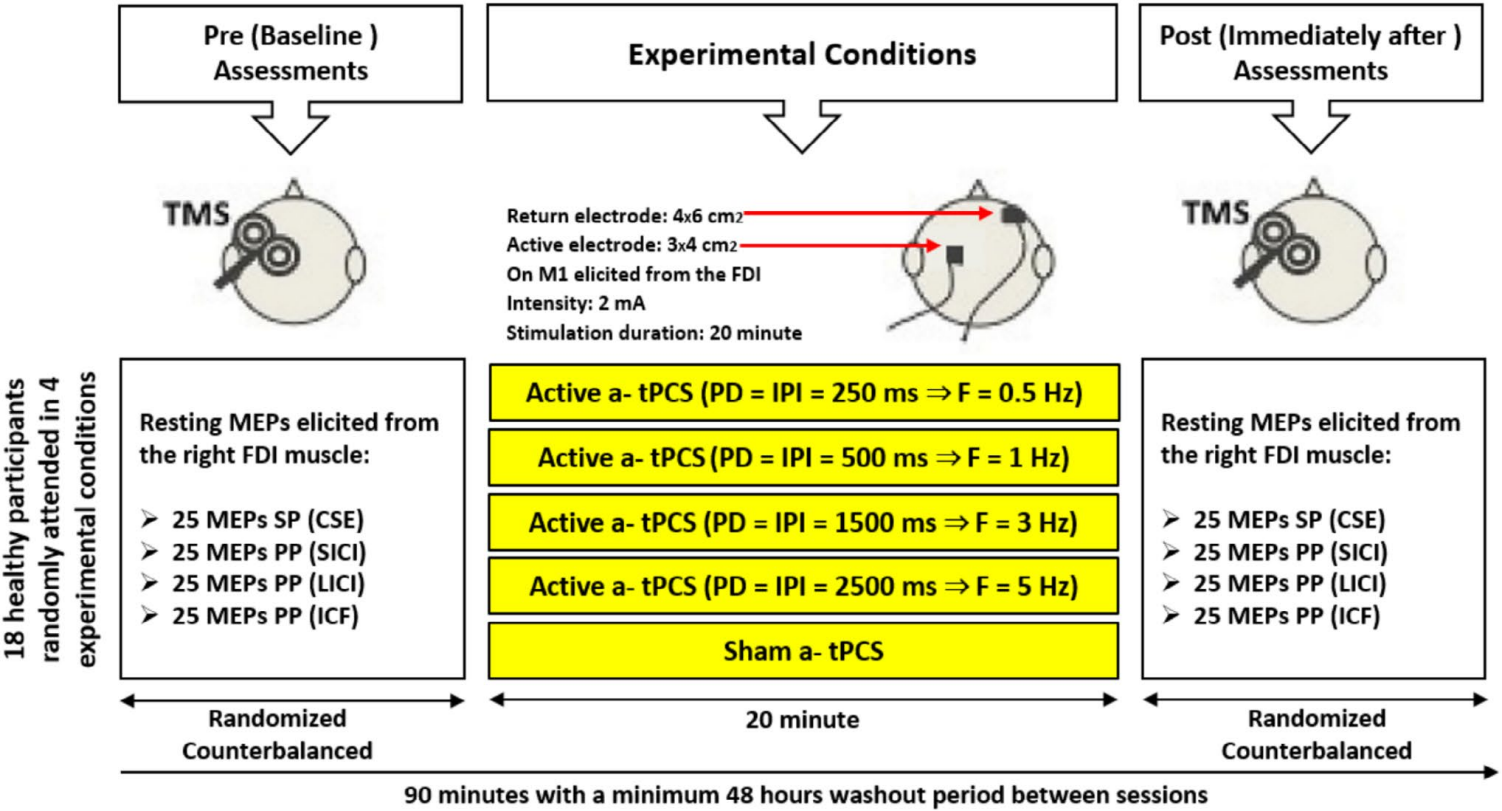
The study design of the effects of a‐tPCS at low frequencies (0.5, 1, 3, 5 Hz, and sham stimulation) on cortical excitability. a‐tPCS, Anodal transcranial pulsed current stimulation; CSE, Corticospinal excitability; F, Frequency; FDI, First dorsal interosseous; Hz, Hertz; ICF, Intra‐cortical facilitation; IPI, Inter‐pulse interval; LICI, Long‐interval intra‐cortical inhibition; M1, Primary left motor cortex; MEPs, Motor evoked potentials; ms, Milliseconds; PD, Pulse duration; PP, Paired‐pulse; SICI, Short‐interval intra‐cortical inhibition; SP, Single‐pulse; TMS, Transcranial magnetic stimulation.

This pilot study utilized a crossover, double‐blinded, sham‐controlled, and randomized design with a minimum 48‐h washout period to mitigate potential carryover effects. This washout period was based on prior tPCS studies, which reported that a single session effect typically lasted only a few hours (Jaberzadeh et al. 2014). All sessions were also conducted at the same time each day to control for diurnal variation. Each participant attended five randomized experimental sessions, receiving 20 min of 2 mA a‐tPCS at one of the following conditions: (1) a‐tPCS at a frequency of 0.5 Hz, (2) a‐tPCS at a frequency of 1 Hz, (3) a‐tPCS at a frequency of 3 Hz, (4) a‐tPCS at a frequency of 5 Hz, and (5) sham a‐tPCS, targeting the M1 of the first dorsal interosseous (FDI) muscle. TMS was applied for pre and immediately post‐intervention assessments within each session. Accordingly, the resting motor threshold (RMT) for TMS‐induced motor‐evoked potentials (MEPs) from the right FDI muscle was measured before and immediately after stimulation. Thus, 25 single‐pulse TMS trials were recorded to assess CSE and 25 paired‐pulse TMS trials were conducted for each protocol evaluating short‐interval intracortical inhibition (SICI), intracortical facilitation (ICF), and long‐interval intracortical inhibition (LICI), to measure inhibitory and facilitatory CCE changes (Figure 1). To control for order effects, the sequence of stimulation conditions was counterbalanced across participants using a randomized method.

Moreover, the assessor responsible for administering the TMS assessments in this study demonstrated reliability through test–retest evaluations conducted during pre‐study training. This study result revealed that the peak‐to‐peak amplitude of MEPs recorded by the same TMS rater at three different time points remained stable, indicating agreement of the Intraclass Correlation Coefficient (ICC) both within (intra‐session) and between (inter‐session) assessments. In this reliability analysis, ICC (3, k) was used, yielding an ICC of 0.85 (95% CI: 0.79–0.98), which reflects good to excellent reliability.

### 2.3 Application of tPCS

#### 2.3.1 Active Stimulation

**FIGURE 2:**
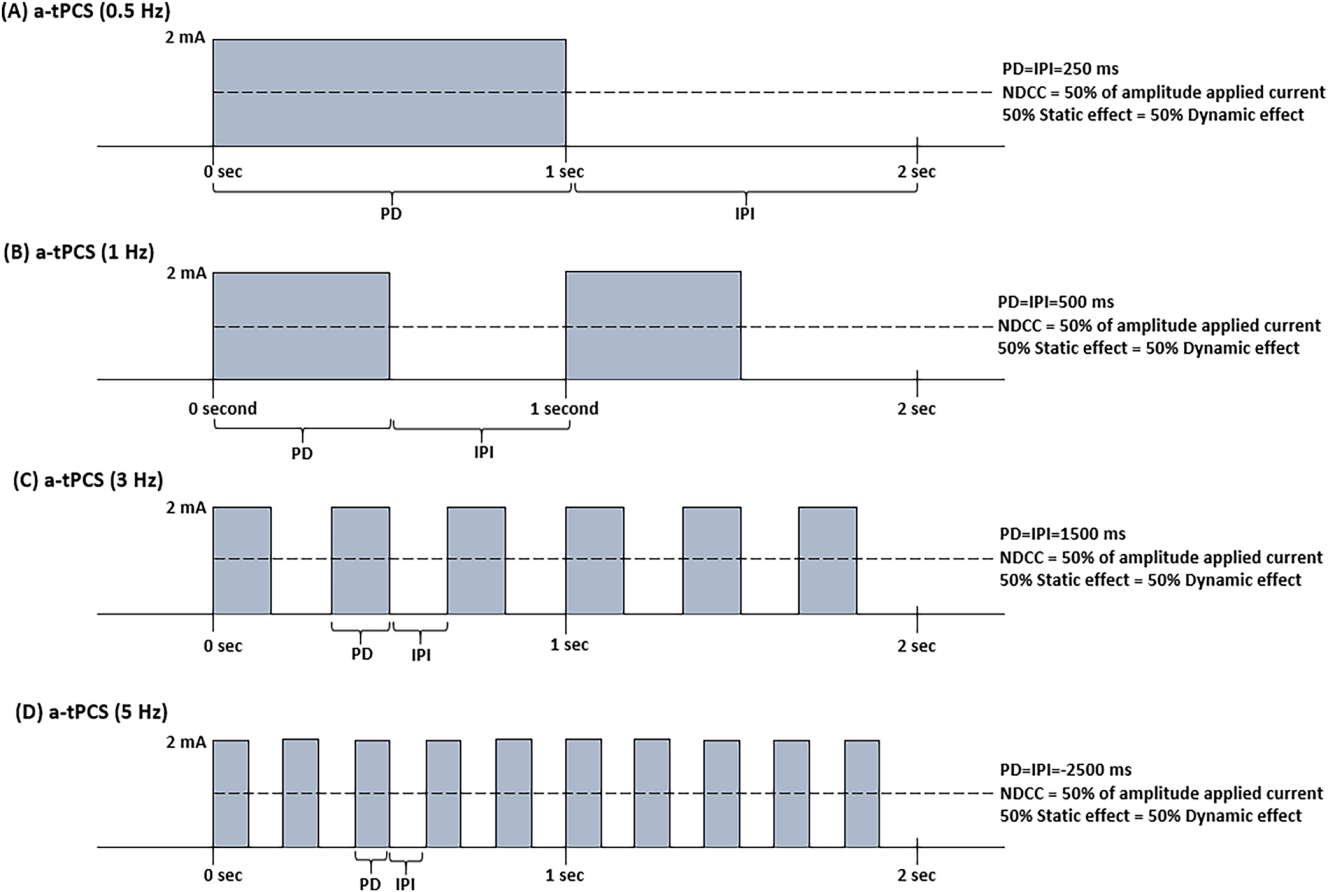
The diagram of the different unidirectional monophasic a‐tPCS including 0.5 Hz frequency of a‐tPCS (A), 1 Hz frequency of a‐tPCS (B), 3 Hz frequency of a‐tPCS (C), 5 Hz frequency of a‐tPCS (D). a‐tPCS, Anodal transcranial pulsed current stimulation; Hz, Hertz; IPI, Inter‐pulse interval; ms, Millisecond; NDCC, Net direct current component; PD, Pulse duration; Sec, Second; SI, Stimulus intensity.

A‐tPCS was delivered via a pair of saline‐soaked surface sponge electrodes by a multichannel brain stimulation system (Setorix Medical HD‐tES, USA). The anode (12 cm^2^) was placed over the M1 of the FDI based on C3 in the 10–20 EEG system, and the cathode (24 cm^2^) was positioned over the contralateral supraorbital area (FP2, per the 10–20 EEG system). Both electrodes were finally secured with two straps. The impedance between the skin and electrodes was automatically monitored and maintained below 10kΩ. The a‐tPCS waveform consisted of unidirectional, rectangular pulses with a 50% duty cycle (PD=IPI), delivered at 2 mA intensity with four frequencies of active a‐tPCS including 0.5 Hz (Figure 2A), 1 Hz (Figure 2B), 3 Hz (Figure 2C), and 5 Hz (Figure 2D) and a sham a‐tPCS. Finally, the total charge (TC) of tPCS in each frequency was automatically calculated using the following formula and maintained throughout all active sessions.

TC is a critical parameter that directly influences the neurophysiological effects of the stimulation.

$$\text{Total charge}\;\left(\mathrm{TC}\right)=\left(\mathrm{SI}\times \mathrm{PD}\times \mathrm{NP}\right)/\mathrm{ES}$$

$$\mathrm{TC}\;\left(0.5\;\mathrm{Hz}\right)=\left(2\;\mathrm{mA}\times 250\;\mathrm{ms}\times 600\;\text{pulses}\right)/12\;{\mathrm{cm}}^{2}=1000\;C/m^{2}$$

$$\mathrm{TC}\;\left(1\;\mathrm{Hz}\right)=\left(2\;\mathrm{mA}\times 500\;\mathrm{ms}\times 1200\;\text{pulses}\right)/12\;{\mathrm{cm}}^{2}=1000\;C/m^{2}$$

$$\mathrm{TC}\;\left(3\;\mathrm{Hz}\right)=\left(2\;\mathrm{mA}\times 1500\;\mathrm{ms}\times 3600\;\text{pulses}\right)/12\;{\mathrm{cm}}^{2}=1000\;C/m^{2}$$

$$\mathrm{TC}\;\left(5\;\mathrm{Hz}\right)=\left(2\;\mathrm{mA}\times 2500\;\mathrm{ms}\times 6000\;\text{pulses}\right)/12\;{\mathrm{cm}}^{2}=1000\;C/m^{2}$$

TC is determined by the stimulus intensity (SI) in mA, the electrode size (ES) in cm^2^, PD in seconds (s), and the number of pulses (NP), which is the product of the PD and the frequency.

#### 2.3.2 Sham Stimulation

In the sham stimulation, electrodes were positioned in the same locations as the experimental conditions in the combination of different frequencies used in this study. However, a‐tPCS was turned off automatically after 30 s (Saavedra et al. 2014) and remained inactive until the final 30 s of the stimulation duration, allowing participants to experience sensations at the beginning and end of the stimulation period. This method ensured participants were unaware of their specific stimulation condition. To assess blinding effectiveness, participants were asked to rate perceived adverse sensations during, immediately, and 30 min after tPCS.

### 2.4 Procedure

#### 2.4.1 Electromyography (EMG)

Participants were seated in an adjustable treatment chair (MagVenture, Denmark) with a headrest and armrests for hand placement and the forearm was rested in the prone position. Then, standard skin preparation was performed, including scraping and cleaning the skin with medical‐grade sandpaper and alcohol wipes, to ensure low skin impedance (≤ 10kΩ) (Gilmore and Meyers 1983; Groppa et al. 2012). TMS‐induced EMG activity from the right FDI muscle was recorded using pre‐gelled self‐adhesive Ag/AgCl surface electrodes (MEDITRACE, Australia) arranged in a belly‐tendon montage. The FDI location was identified using anatomical landmarks and confirmed by muscle contraction during a card‐holding test, while the ground electrode was placed on the styloid process of the ulnar bone (Oh 2003). Electrodes were secured with hypoallergenic tape, and accurate placement was verified by monitoring real‐time EMG activity. EMG signals were amplified (×1000), band‐pass filtered (10–500 Hz), sampled at 1000 Hz, and processed online using LabChart 8 software (AD Instruments, Australia) via a laboratory analogue‐to‐digital interface (Scope View: PowerLab 8/30; AD Instruments).

#### 2.4.2 Transcranial Magnetic Stimulation (TMS)

Biphasic single and paired‐pulse TMS stimuli were delivered using a MagPro R30 stimulator (MagVenture, Denmark) with a figure‐of‐eight coil (max. initial dB/dt 28 KT/s). The coil was positioned tangentially over the left hemisphere at a 45° angle from the midline sagittal skull plane, with the handle oriented posterior‐to‐anterior (Vaseghi et al. 2015). The hot spot in the left M1 region, eliciting the strongest MEPs from the right FDI muscle, was identified and marked for the remaining testing. TMS pulses were applied at 0.1–0.2 Hz to prevent long‐lasting M1 excitability changes (Chen, Gerloff, et al. 1997). RMT was determined using the parameter estimation sequential testing (PEST) adaptive threshold‐hunting method, where an MEP amplitude > 50 μV was considered a successful trial (Awiszus 2003; Silbert et al. 2013). RMT measurements adhered to safety guidelines with 95% confidence intervals (Julkunen et al. 2012). Each pre‐ and post‐assessment included an average of 25 single‐pulse and 75 paired‐pulse TMS trials with varying inter‐stimulus intervals (ISIs) (Biabani et al. 2017), recorded using the LabChart software (PowerLab 8/30, ADInstruments, Australia) and stored for offline analysis. Finally, MEPs with any feature exceeding two standard deviations from the mean were identified as outliers and removed (Nguyen et al. 2019; Schilberg et al. 2021).

##### 2.4.2.1 Single‐Pulsed TMS

Single‐pulse TMS was applied to assess CSE changes. In this protocol, the peak‐to‐peak amplitude of MEP indicates a significant correlation with glutamate levels within the motor cortex (Stagg and Nitsche 2011). A smaller MEP amplitude indicated lower CSE, while a higher amplitude suggested increased excitability. Therefore, the average of 25 MEPs was recorded using an intensity set at 120% of the RMT and a 6 s ISI (Vaseghi et al. 2015; Behrangrad et al. 2022). This intensity and ISI were also consistently maintained during the post‐assessment within a session to compare the differences between pre‐ and post‐CSE.

##### 2.4.2.2 Paired‐Pulsed TMS

Paired‐pulse TMS was also employed to assess the CCE. These protocols included inhibitory and facilitatory mechanisms using two pulses with different ISIs. The first TMS pulse was a conditioned stimulus (CS) paired with a second pulse or test stimulus (TS). As the procedure of post‐protocols was similar to pre‐ones, CS and TS were set with adjusted intensities based on post‐RMT measurements to compare the intervention effects in each session.

##### 2.4.2.3 ICF And SICI

The ICF protocol was used to assess excitatory neurotransmission primarily mediated by glutamatergic receptor activity. In this protocol, the CS, set at 80% RMT (0.8 x RMT), was paired with a supra‐threshold TS (at an intensity to induce ∼1 mV MEPs with an ISI of 10 ms). This measure reflects synaptic facilitation, likely involving NMDA receptor activity. The SICI protocol was applied to evaluate inhibitory processes associated with GABA_A_ receptor activity. Using a conditioning stimulus at 80% RMT, followed by a supra‐threshold test stimulus at an ISI of 3 ms, this protocol quantifies fast‐acting intracortical inhibition mediated by GABAergic neurotransmission (Kujirai et al. 1993).

##### 2.4.2.4 LICI

The LICI protocol was then applied by two supra‐threshold pulses (CS and TS) adjusted to produce a peak‐to‐peak amplitude of ∼1 mV with an ISI of 150 ms. This protocol is mediated by slower‐acting metabotropic (GABA_B_) receptors that play a crucial role in motor function (Mcdonnell et al. 2006).

### 2.5 Measurement of the Side Effects

All participants completed a form to assess the potential side and adverse effects of low‐frequency a‐tPCS reported in different experimental conditions. These side effects included tingling, itching, headaches, phosphenes, and burning sensations under both active and return electrodes (Dissanayaka et al. 2020, 2022). Participants rated these sensations on a numeric analogue scale (NAS), with 0 indicating none, 1–3 mild, 4–6 moderate, and 7–10 severe. Sensations were recorded at baseline, during stimulation (at 0–2, 6–8, 12–14, and 18–20 min), immediately post‐stimulation, and 30 min afterwards. To minimize expectation bias, participants were provided with standardized, neutral information about possible sensations without specifying whether these were more likely to occur in active or sham conditions. Blinding integrity was also assessed by asking participants at the end of the intervention to indicate their perception of the stimulation as ‘active stimulation,’ ‘sham stimulation,’ or ‘cannot say’

### 2.6 Study Variables

This study employed a within‐subjects design with two independent variables: experimental conditions and time. The experimental conditions variable consisted of five levels (0.5, 1, 3, 5 Hz, and sham stimulation), while the time variable had two levels (pre and post‐intervention). The dependent variables included CSE, ICF, SICI, LICI, and side effects (itching, tingling, burning, headache, and phosphene) reported during tPCS. The study aimed to evaluate the impact of the experimental conditions and their interaction with time on these dependent variables.

### 2.7 Data Management and Statistical Analysis

Statistical analyses were conducted using GraphPad Prism software (version 10). The Shapiro–Wilk test was applied to assess the normality of the data. The Chi‐square test was used to evaluate the effectiveness of blinding by analyzing participants' responses regarding their perception of the stimulation condition. A one‐way repeated‐measures ANOVA was used to check for baseline values across the five a‐tPCS conditions. Bonferroni‐corrected post hoc paired‐sample t‐tests were conducted to compare baseline values across all five experimental conditions and exclude potential carryover effects. Separate two‐way repeated‐measures ANOVA tests (GLM‐based) were conducted for each outcome measure (CSE, SICI, ICF, LICI, RMT, and side effects) to evaluate the impacts of a‐tPCS. Experimental sessions (0.5, 1, 3, 5 Hz, and sham) and time (pre‐ and post‐intervention) were included as within‐subject factors for each outcome. Post hoc pairwise comparisons with Bonferroni correction were also conducted for multiple comparisons. Statistical significance was set at *p* < 0.05 for all analyses, and results were reported with corresponding *p*‐values and F‐statistics to ensure clarity in interpretation.

## 3 Results

### 3.1 Participants

The study involved 18 healthy participants, recruited from university students and community volunteers. Inclusion criteria included ages 18–45 and no medical conditions. In this crossover design, participants underwent five stimulation conditions with ≥ 48‐h washouts to minimize carryover effects. They were randomly assigned to start with one condition, and baseline comparisons confirmed balanced groups. The study received ethics approval (ID: 32335) from the Monash University Human Research Ethics Committee, and all participants provided written informed consent after being informed of the study's purpose and procedures. Confidentiality was maintained by anonymizing and securely storing data, accessible only to authorized researchers, by the Declaration of Helsinki and the National Statement on Ethical Conduct (1964).

### 3.2 Blinding Integrity

Participants were asked about their beliefs regarding the type of stimulation they received (active, sham, or cannot say) immediately after stimulation. The Chi‐square test revealed no significance (χ^2^ (8) = 7.24, *p* = 0.51), indicating that participants could not reliably distinguish between the types of stimulation received, confirming successful blinding. Overall, 27.77% of participants accurately guessed the stimulation, while 72.23% did not (including those who responded ‘cannot say’ or ‘sham’ in the active stimulation groups) (Table 1).

**TABLE 1:** Number of participants who correctly guessed the active or sham stimulation conditions.

Experimental conditions (*n* = 18)								
		0.5 Hz	1 Hz	3 Hz	5 Hz	Sham	Total	Correct guess
Perceived stimulation	Active	5	7	5	3	3	23	20
	Sham	5	1	4	7	5	22	5
	Cannot say	8	10	9	8	10	45	0
	Total	18	18	18	18	18	90	25

### 3.3 Baseline Measurements

#### 3.3.1 TMS Baseline

One‐way ANOVA revealed no significant differences in baseline MEP amplitudes of CSEs (F (4, 85) = 0.50, *p* = 0.73), ICF (F (4, 85) = 0.31, *p* = 0.86), SICI (F (4, 85) = 0.52, *p* = 0.71), and LICI (F (4, 85) = 0.50, *p* = 0.72) across the five experimental conditions. These findings indicate that baseline neurophysiological measures were consistent across all conditions, confirming no carryover effects between sessions and ensuring that any observed effects in later phases are not due to baseline differences.

#### 3.3.2 RMT Baseline

One‐way ANOVA revealed no significant differences in baseline RMT (F (4, 85) = 0.33, *p* = 0.85), ensuring no carryover effects between sessions.

#### 3.3.3 Side Effects Baseline

One‐way ANOVA revealed no significant differences in baseline side effects, including phosphine, burning, itching, tingling, and headache, before stimulation (F (16, 340) = 1, *p* = 0.45). Therefore, any adverse effects observed during or after stimulation can be attributed to the application of currents.

### 3.4 Effects of Low Frequencies of tPCS on CSE

**FIGURE 3:**
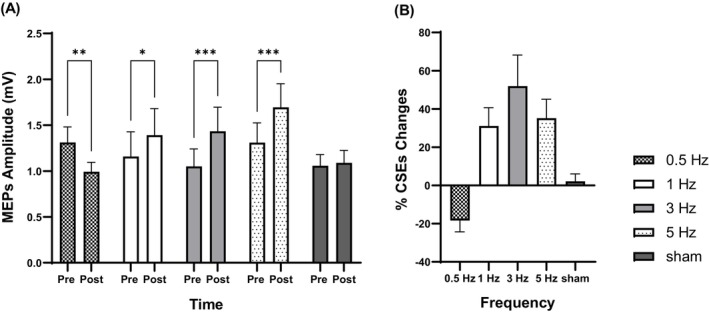
Effects of a‐tPCS at low frequencies (0.5, 1, 3, and 5 Hz) and sham stimulation CSE (A). The percentage of changes a‐tPCS at low frequencies (0.5, 1, 3, and 5 Hz) and sham stimulation on CSE (B). *p < 0.05; **p < 0.01; ***p < 0.001. CSE: Corticospinal excitability; a‐tPCS: Anodal transcranial pulse current stimulation.

The results demonstrated that low frequencies of active a‐tPCS significantly altered CSE immediately after stimulation, whereas sham stimulation did not produce significant changes (Figure 3A).

The two‐way ANOVA ((2 time points, pre vs. post) × 5 stimulation conditions) for CSE outcome measures revealed a significant interaction effect (stimulation frequency × time) for all experimental conditions F ((4, 85) = 7.18, *p* < 0.0001), except for sham (*t* (85) = 0.28, *p* = 0.77). Post hoc pairwise comparisons were conducted using the Bonferroni correction.

#### 3.4.1 One‐Half Hz

A‐tPCS at 0.5 Hz applied to M1 significantly decreased CSE (*t* (85) = 2.88, *p* = 0.0049) with an 18.40% reduction in MEP amplitude from pre‐ to post‐stimulation (Figure 3B), whereas no such change was observed in the sham condition.

#### 3.4.2 One, Three, and Five Hz

Application of A‐tPCS over M1 at 1 Hz (*t* (85) = 2.11, *p* = 0.037), 3 Hz (*t* (85) = 3.46, *p* = 0.0008) Hz, and 5 Hz (*t* (85) = 3.49, *p* = 0.0008) resulted in a significant increase in CSE compared to sham stimulation. The percentage changes in MEP amplitude from pre‐ to post‐stimulation were as follows: 31.09%, 51.91%, and 35.19% for the 1 Hz, 3 Hz, and 5 Hz conditions, respectively, whereas the sham condition showed a negligible change of 2.15% (Figure 3B).

### 3.5 Effects of Low Frequencies of tPCS on ICF, SICI, and LICI

#### 3.5.1 One‐Half Hz

**FIGURE 4:**
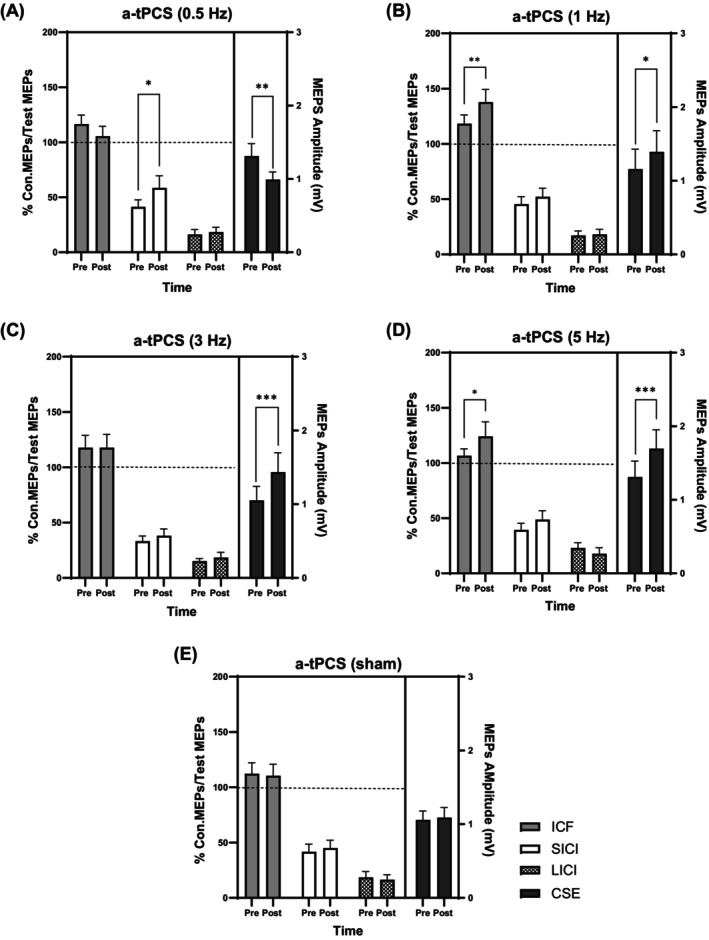
Effects of low frequencies a‐tPCS on CSE and CCE. The effects of a‐tPCS (0.5, 1, 3, and 5 Hz) on ICF, SICI, and LICI compared to sham stimulation. The dotted line at the ratio of 100% represents a baseline or control level of motor evoked potential (MEP) amplitude. This line serves as a reference point, indicating the level of MEP response without any inhibitory or facilitatory conditioning stimulus applied. *p < 0.05; **p < 0.01; ***p < 0.001. CCE, Corticocortical excitability; Con., Conditioned test of averaged 25 MEPs; CSE, Corticospinal excitability; ICF, Intracortical facilitation; LICI, Long interval intracortical inhibition; MEPs, Motor evoked potential; SICI, Short interval intracortical inhibition; Test MEP, Averaged of 25 MEPs 1mv.

A repeated‐measures ANOVA indicated a significant increase in SICI following a‐tPCS at 0.5 Hz (*t* (68) = 2.56, *p* = 0.012). Paired t‐tests showed a 44.95% increase in inhibition mediated by GABA_A_ receptors from pre‐ to post‐stimulation. This finding indicates that a‐tPCS at 0.5 Hz modulates intracortical circuits involved in inhibitory control. In contrast, no significant changes were observed in ICF (*t* (68) = 1.74, *p* = 0.10) or LICI (*t* (68) = 0.34, *p* = 0.73) following a‐tPCS at 0.5 Hz, suggesting that excitatory intracortical circuits and GABA_B_‐mediated inhibition remained unaffected, respectively (Figure 4A). Therefore, an increase in SICI without changes in ICF and LICI leads to a reduction in CSE.

#### 3.5.2 One and five Hz

A repeated‐measures ANOVA indicated a significant increase in ICF following a‐tPCS at 1 Hz (*t* (68) = 2.81, *p* = 0.0064) and 5 Hz (t (68) = 2.59, *p* = 0.011). Paired t‐tests showed a 23.83% increase at 1 Hz (Figure 4B) and a 16.85% increase at 5 Hz (Figure 4D) from pre‐ to post‐stimulation, suggesting enhancement of glutamate‐mediated excitatory intracortical circuits in the motor cortex. In contrast, no significant changes were found in GABAergic‐mediated inhibition, as measured by SICI (*t* (68) = 0.95, *p* = 0.34) or LICI (*t* (68) = 0.13, *p* = 0.89) at 1 Hz and SICI (*t* (68) = 1.39, *p* = 0.16) or LICI (*t* (68) = 0.77, *p* = 0.43) following 5 Hz stimulation. An increase in ICF was observed, while SICI and LICI remained unchanged. This was accompanied by higher CSE measures.

#### 3.5.3 Three Hz and Sham

The results involved pre‐ and post‐stimulation comparison within each condition of 3 Hz and sham. There were no significant changes in ICF (*t* (68) = 0.005, *p* = 0.99), SICI (*t* (68) = 0.89, *p* = 0.37), and LICI (*t* (68) = 0.59, *p* = 0.55) following a‐tPCS of 3 Hz (Figure 4C). Similarly, sham stimulation did not influence ICF (*t* (68) = 0.31, *p* = 0.75), SICI (*t* (68) = 0.60, *p* = 0.54), and LICI (*t* (68) = 0.37, *p* = 0.70) (Figure 4E), indicating that excitatory and inhibitory intracortical circuits remained unaffected by both conditions. Therefore, no significant changes were observed in SICI, ICF, or LICI, and CSE remained unaltered.

Descriptive statistics, including means, SD, minimum and maximum values, of raw data were also reported for all dependent variables (CSE, SICI, ICF, and LICI) across all stimulation conditions (0.5, 1, 3, 5 Hz, and sham), and for both pre‐ and post‐intervention time points (Table 2).

**TABLE 2:** Descriptive Statistics of data.

Variables	Conditions	Means		SD		Min		Max	
		Pre (mV)	Post (mV)	Pre	Post	Pre (mV)	Post (mV)	Pre (mV)	Post (mV)
CSE	0.5 Hz	1.30	0.96	0.66	0.33	0.62	0.37	2.52	1.45
	1 Hz	1.28	1.48	1.36	1.43	0.37	0.35	5.24	5.31
	3 Hz	1.12	1.52	0.88	1.30	0.41	0.50	3.23	5.95
	5 Hz	1.36	1.72	0.97	1.77	0.52	0.68	4.97	5.87
	Sham	1.32	1.30	1.16	1.03	0.53	0.57	5.49	4.48
SICI	0.5 Hz	40.68	53.65	27.09	40.03	14.10	10.71	118.67	143.08
	1 Hz	51.09	48.16	29.10	29.81	13.09	13.24	106.37	96.75
	3 Hz	40.73	34.48	21.78	23.47	10.59	8.74	91.79	79.81
	5 Hz	46.03	39.67	27.40	26.02	13.14	11.98	102.36	110.67
	Sham	54.71	61.59	53.16	63.60	20.29	6.86	230.66	290.31
ICF	0.5 Hz	116.80	110.81	23.17	35.48	65.23	48.89	176.40	171.25
	1 Hz	128.61	139.93	38.93	48.07	69.05	48.07	192.89	265.67
	3 Hz	127.76	117.05	55.17	47.74	63.53	56.39	264.93	265.75
	5 Hz	113.37	126.47	32.27	43.74	55.76	50.74	175.42	209.9
	Sham	127.84	122.61	52.04	66.57	61.55	59.38	264.29	347.97
LICI	0.5 Hz	15.82	23.05	23.83	22.25	1.73	2.25	101.40	107.78
	1 Hz	19.47	19.79	21.78	23.72	3.07	2.40	75.69	88.91
	3 Hz	16.65	23.11	14.06	29.76	3.94	2.83	55.26	107.129
	5 Hz	18.38	13.24	16.32	16.58	2.58	1.29	58.13	71.96
	Sham	21.62	23.64	26.23	27.34	3.18	1.54	96.09	76.12

### 3.6 Adverse Effects of Low Frequencies of tPCS

The two‐way repeated measures ANOVA revealed no headaches (*p* < 0.05) during or after current stimulation. Significant sensations, including phosphene, itching, tingling, and burning, were observed during stimulation, but all participants tolerated the experimental conditions without procedural interruptions due to adverse effects. Also, no significant sensations (*p* > 0.999) were reported immediately or 30 min post‐stimulation.

#### 3.6.1 One‐Half Hz

The findings at 0.5 Hz indicated significant side effects (*p* < 0.05), including sensations of burning, itching, and tingling under the active electrode during the first 8 min of stimulation (Table 3).

**TABLE 3:** Side effects of low frequencies of a‐tPCS.

Stimulation	Sensation	Electrode	*p*‐value (*α* < 0.05)						
			Time (minute)						
			Pre	0–2	6–8	12–14	18–20	Immediately after	30‐min after
a‐tPCS (0.5 Hz)	Phosphene	Active	Baseline	N (0.9966)	N (0.8883)	N (0.5353)	N (0.8883)	N (0.999)	N (0.999)
		Return		N (0.9966)	N (0.999)	N (0.999)	N (0.999)	N (0.999)	N (0.999)
	Burning	Active		Y (0.0001****)	Y (0.0002***)	N (0.3605)	N (0.9074)	N (0.999)	N (0.999)
		Return		N (0.0819)	N (0.5874)	N (0.9674)	N (0.9992)	N (0.999)	N (0.999)
	Itching	Active		Y (0.0003***)	Y (0.0337*)	N (0.0538)	N (0.0538)	N (0.9989)	N (0.999)
		Return		N (0.913)	N (0.913)	N (0.9948)	N (0.999)	N (0.999)	N (0.999)
	Tingling	Active		Y (0.0001****)	Y (0.0058**)	N (0.0749)	N (0.2361)	N (0.999)	N (0.999)
		Return		N (0.0749)	N (0.6425)	N (0.9072)	N (0.9816)	N (0.999)	N (0.999)
	Headache	Active		N (0.999)	N (0.9499)	N (0.8249)	N (0.3970)	N (0.999)	N (0.999)
		Return		N (0.999)	N (0.999)	N (0.999)	N (0.999)	N (0.999)	N (0.999)
a‐tPCS (1 Hz)	Phosphene	Active	Baseline	N (0.1132)	N (0.1954)	N (0.9215)	N (0.7003)	N (0.999)	N (0.999)
		Return		Y (0.0001****)	Y (0.0001****)	Y (0.0001****)	Y (0.0001****)	N (0.999)	N (0.999)
	Burning	Active		Y (0.0001****)	Y (0.0006***)	Y (0.0079**)	Y (0.0139*)	N (0.999)	N (0.999)
		Return		Y (0.0012**)	Y (0.0006***)	Y (0.0237*)	N (0.9499)	N (0.999)	N (0.999)
	Itching	Active		Y (0.0016**)	Y (0.0282*)	N (0.2312)	N (0.6375)	N (0.999)	N (0.999)
		Return		N (0.5253)	N (0.1105)	N (0.3162)	N (0.5253)	N (0.999)	N (0.999)
	Tingling	Active		Y (0.0001****)	Y (0.0001****)	Y (0.0043**)	Y (0.0003***)	N (0.999)	N (0.999)
		Return		Y (0.0003***)	Y (0.0233*)	Y (0.0006***)	Y (0.0233*)	N (0.999)	N (0.999)
	Headache	Active		N (0.999)	N (0.9951)	N (0.9840)	N (0.9840)	N (0.999)	N (0.999)
		Return		N (0.7713)	N (0.5644)	N (0.7713)	N (0.999)	N (0.999)	N (0.999)
a‐tPCS (3 Hz)	Phosphene	Active	Baseline	N (0.2762)	N (0.2195)	N (0.3409)	N (0.4892)	N (0.999)	N (0.999)
		Return		Y (0.0001****)	Y (0.0001****)	Y (0.0001****)	Y (0.0004***)	N (0.999)	N (0.999)
	Burning	Active		Y (0.0001****)	Y (0.0242*)	N (0.8120)	N (0.9691)	N (0.999)	N (0.999)
		Return		Y (0.0383*)	N (0.2482)	N (0.3288)	N (0.3288)	N (0.999)	N (0.999)
	Itching	Active		Y (0.0001****)	Y (0.0023**)	Y (0.0320*)	N (0.2267)	N (0.999)	N (0.999)
		Return		N (0.9865)	N (0.9584)	N (0.9584)	N (0.9584)	N (0.999)	N (0.999)
	Tingling	Active		Y (0.0049**)	N (0.1295)	N (0.0928)	N (0.4701)	N (0.999)	N (0.999)
		Return		Y (0.0049**)	Y (0.0003***)	Y (0.0299*)	N (0.3828)	N (0.999)	N (0.999)
	Headache	Active		N (0.9984)	N (0.999)	N (0.999)	N (0.9392)	N (0.9984)	N (0.999)
		Return		N (0.9984)	N (0.9984)	N (0.9853)	N (0.9392)	N (0.9984)	N (0.999)
a‐tPCS (5 Hz)	Phosphene	Active	Baseline	N (0.4017)	N (0.4017)	N (0.4607)	N (0.8117)	N (0.999)	N (0.999)
		Return		Y (0.0001****)	Y (0.0001****)	Y (0.0001****)	Y (0.0006***)	N (0.999)	N (0.999)
	Burning	Active		N (0.3401)	N (0.1135)	N (0.6854)	N (0.9984)	N (0.999)	N (0.999)
		Return		Y (0.0045**)	Y (0.0045**)	N (0.2462)	N (0.4496)	N (0.999)	N (0.999)
	Itching	Active		Y (0.0009***)	Y (0.0035**)	Y (0.0035**)	Y (0.0218*)	N (0.999)	N (0.999)
		Return		N (0.6645)	N (0.5436)	N (0.4241)	N (0.4241)	N (0.999)	N (0.999)
	Tingling	Active		Y (0.0064**)	N (0.1957)	Y (0.0042**)	N (0.1503)	N (0.999)	N (0.999)
		Return		N (0.2498)	N (0.0839)	N (0.3830)	N (0.3830)	N (0.999)	N (0.999)
	Headache	Active		N (0.999)	N (0.999)	N (0.999)	N (0.999)	N (0.999)	N (0.9986)
		Return		N (0.9451)	N (0.8763)	N (0.9151)	N (0.7734	N (0.9986)	N (0.9986)
a‐tPCS (Sham)	Phosphene	Active	Baseline	N (0.999)	N (0.999)	N (0.999)	N (0.999)	N (0.999)	N (0.999)
		Return		N (0.1172)	N (0.999)	N (0.999)	N (0.999)	N (0.999)	N (0.999)
	Burning	Active		N (0.9915)	N (0.999)	N (0.999)	N (0.5556)	N (0.999)	N (0.999)
		Return		N (0.3279)	N (0.9998)	N (0.9998)	N (0.1616)	N (0.999)	N (0.999)
	Itching	Active		Y (0.0003***)	N (0.999)	N (0.999)	Y (0.0001****)	N (0.999)	N (0.999)
		Return		N (0.2928)	N (0.999)	N (0.9979)	N (0.8106)	N (0.999)	N (0.999)
	Tingling	Active		Y (0.0001****)	N (0.999)	N (0.999)	Y (0.0048**)	N (0.999)	N (0.999)
		Return		N (0.999)	N (0.999)	N (0.999)	N (0.999)	N (0.999)	N (0.999)
	Headache	Active		N (0.999)	N (0.999)	N (0.999)	N (0.999)	N (0.999)	N (0.999)
		Return		N (0.5018)	N (0.5018)	N (0.999)	N (0.999)	N (0.999)	N (0.999)

*Note:* The side effects of a‐tPCS on different high frequencies (10, 25, and 80 Hz) to sham stimulation, including: Phosphene, burning, itching, tingling, and headache. N means that no side effects, and Y means the presence of side effects. *p < 0.05; **p < 0.01; ***p < 0.001; ****p < 0.0001 from low significance to high significance.

#### 3.6.2 One Hz

Findings at 1 Hz revealed significant side effects (*p* < 0.05) of burning and itching under both active and return electrodes, and phosphene under the return electrode throughout the stimulation period. The tingling was also significant under the active electrode during the first 8 min (Table 3).

#### 3.6.3 Three Hz

Findings of 3 Hz showed significant (*p* < 0.05) phosphene and tingling sensation under the return electrode, and itching under the active electrode throughout the stimulation period. Burning was significant under the active electrode during the first 8 min and under the return electrode during the first 2 min, while tingling under the active electrode was significant for the first 2 min (Table 3).

#### 3.6.4 Five Hz

At 5 Hz, significant side effects (*p* < 0.05) indicated phosphene under the active electrode and itching under the return electrode throughout the stimulation period. Burning was significant under the return electrode during the first 8 min, while tingling under the active electrode was significant during the first 2 min and between 12 to 14 min (Table 3).

#### 3.6.5 Sham

During sham stimulation, significant itching and tingling (*p* < 0.05) were observed under the active electrode during the first 2 min and the final 2 min of stimulation (Table 3).

## 4 Discussion

This study examined the effects of low frequencies a‐tPCS (0.5, 1, 3, and 5 Hz) over M1 on key neurophysiological measures, including CSE, SICI, LICI, and ICF. Consistent with prior research, sham stimulation did not alter CSE or CCE, confirming its lack of impact on CE (Behrangrad et al. 2022). These findings underscore the role of experimental stimulation in modulating cortical activity and eliciting neurophysiological responses.

### 4.1 Effects of Low Frequencies of tPCS on CSE

#### 4.1.1 One‐Half Hz

The findings revealed a significant decrease in CSE following 0.5 Hz a‐tPCS, consistent with a previous a‐tPCS study indicating that similar stimulation inhibited neural fluctuations via LTD (Luu et al. 2016).

#### 4.1.2 One, Three, and Five Hz

These frequencies of a‐tPCS significantly increased CSE, aligning with a prior study on 4 Hz a‐tPCS (Dissanayaka et al. 2022). Notably, the percentage changes for 1 and 5 Hz were similar (31% and 35%, respectively), while 3 Hz demonstrated the most pronounced effect, with a 52% change.

Based on these findings, it is reasonable to anticipate that tPCS may produce effects similar to those observed with TMS. Studies investigating the effects of TMS on MEPs report that low‐frequency stimulation (< 1 Hz) induces intracortical inhibition, whereas higher frequencies (≥ 5 Hz) are associated with cortical facilitation (Chen, Classen, et al. 1997; Fitzgerald et al. 2006). Given the similarities in their frequency‐dependent modulation of CE, it is plausible to hypothesize that tPCS could exert comparable effects.

However, despite these parallels, the underlying biophysical mechanisms of tPCS and TMS differ significantly. TMS directly induces action potentials via electromagnetic induction, leading to immediate neuronal depolarization and synaptic activation (Terao and Ugawa 2002). In contrast, tPCS is thought to modulate neural activity through subthreshold oscillatory entrainment, where weak electric fields influence neuronal membrane potentials without directly triggering action potentials. Entrainment refers to the synchronization of neural oscillations with the externally applied stimulation frequency, which can modify brain network dynamics and potentially influence CE. This entrainment may alter CE by promoting resonance effects and modifying ongoing endogenous rhythms (Vasquez et al. 2016; Thibaut et al. 2017). As a result, while both techniques show frequency‐dependent effects, their modes of action are distinct.

Thus, while tPCS holds promise as a novel approach for modulating neural processes and addressing various clinical conditions, further research is required to clarify the extent of the mechanistic similarities and differences between these two neurostimulation techniques.

### 4.2 Effects of Low Frequencies of tPCS on ICF, SICI, and LICI

#### 4.2.1 One‐Half Hz

The findings indicated that 0.5 Hz of a‐tPCS significantly increased SICI, reflecting enhanced GABA_A_ receptor‐mediated inhibitory activity in the cortex (Behrangrad et al. 2022). This suggests that extremely low‐frequency a‐tPCS may preferentially engage fast‐acting, synaptic inhibition mediated by GABA_A_, contributing to reduced CE.

#### 4.2.2 One and five Hz

A‐tPCS at 1 and 5 Hz significantly increased ICF, indicating enhanced intracortical facilitation likely mediated by glutamate activity. This aligns with prior findings on TMS‐induced excitatory plasticity, where ≥ 5 Hz stimulation enhances CE via NMDA‐receptor‐dependent mechanisms (Oliveri et al. 2005). The increased ICF suggests that low‐frequency alternating currents may modulate synaptic transmission, potentially by promoting LTP‐like effects in excitatory networks.

#### 4.2.3 Three Hz

Although 3 Hz a‐tPCS significantly increased CSE, it did not induce significant LICI, SICI, or ICF changes. This suggests that mechanisms beyond classic GABA_A_ or glutamatergic plasticity may contribute to CE at this frequency. Thus, the neuromodulatory effects may involve network‐level oscillatory entrainment rather than direct synaptic modulation.

In summary, these findings suggest that the mechanisms underlying 0.5 to 5 Hz frequencies of a‐tPCS extend beyond GABA_A_‐mediated (< 1 Hz) and glutamatergic (≥ 1 Hz) responses. The lack of significant LICI modulation further highlights potential differences in how a‐tPCS and TMS engage GABA_B_‐mediated inhibition. Future studies using pharmacological interventions or EEG‐TMS measures of oscillatory activity could provide deeper insights into the specific neural circuits affected by 0.5 to 5 Hz frequencies of a‐tPCS.

### 4.3 Adverse Effects of Low Frequencies of tPCS

Neuromodulation methods are generally safe for healthy volunteers. However, tDCS studies commonly reported itching and tingling as adverse effects, with headaches and burning sensations occurring less frequently (Brunoni et al. 2011). Consistent with previous studies (Jaberzadeh et al. 2014, 2015), the current study observed itching, tingling, and burning sensations with 0.5 to 5 Hz frequencies a‐tPCS, while a‐tPCS (≥ 1 Hz) induced phosphene effects during stimulation. All side effects resolved immediately after stimulation.

#### 4.3.1 One‐Half Hz

This study suggested that 0.5 Hz a‐tPCS is a safe form of stimulation, causing only mild effects like itching, tingling, and burning under the active electrode, with no phosphene effects.

#### 4.3.2 One Hz

Findings indicated that 1 Hz a‐tPCS causes phosphene effects, itching, tingling, and burning sensations under both electrodes, similar to tDCS (Brunoni et al. 2011).

#### 4.3.3 Three Hz

The adverse effects of 3 Hz a‐tPCS were milder than those of 1 Hz, including itching, tingling under the return electrode, and brief burning sensations under the active electrode.

#### 4.3.4 Five Hz

The adverse effects of 5 Hz a‐tPCS were milder than those of 1 and 3 Hz, including brief itching under the active electrode, tingling, and burning sensations under the return electrode.

#### 4.3.5 Sham

This stimulation caused brief itching and tingling under both electrodes for 2 min at the beginning and end of stimulation, aligning with previous studies designed for blind participants (Brunoni et al. 2011).

In summary, the findings indicated that a‐tPCS (< 1 Hz) had minimal side effects, and side effects decreased as the frequency increased beyond 1 Hz. However, phosphene was identified as the primary adverse effect during a‐tPCS (≥ 1 Hz). According to previous studies, the adjustable IPI, PD, and frequency in tPCS allow for varying NDCC, thereby minimizing the electrochemical effects caused by acidic and basic reactions under the electrodes (Jaberzadeh et al. 2014, 2015). This combination of dynamic and static effects distinguishes tPCS from other neuromodulatory techniques, enabling better control of side effects and the development of personalized protocols (Malekahmad et al. 2024).

### 4.4 Limitations and Suggestions for Future Research

This study offers important insights into the effects of low frequencies of a‐tPCS on CSE and CCE; however, several limitations warrant consideration. First, this study did not address potential gender differences in responses to tPCS despite evidence that sex hormones influence CE and neuroplasticity (Pitcher et al. 2003).

Second, the findings are constrained by the predominance of young adult participants, limiting their applicability to older populations, where age‐related changes in CSE and CCE may play a significant role (Pitcher et al. 2003).

Third, the research focused solely on the immediate aftereffects of a‐tPCS, leaving the long‐term effects on neuroplasticity unexplored. Longitudinal studies are required to ascertain the persistence of these changes over time.

Fourth, the neural entrainment mechanism hypothesized to be induced by tPCS should be examined using quantitative EEG on coherency brainwaves.

Additionally, future studies could explore dose–response relationships, where different stimulation durations and intensities are tested to determine the optimal parameters for modulating CE and achieving therapeutic effects. These dose–response studies could provide valuable insights into the efficacy and safety of tPCS across varying levels of stimulation, helping to fine‐tune its application for clinical use. While the crossover design improves statistical power by reducing inter‐individual variability, the relatively small sample size (*n* = 18) may limit the generalizability of the findings, particularly to clinical populations. Future studies with larger and more diverse samples, including individuals with clinical conditions, are needed to confirm the broader applicability of the results.

Furthermore, while this study underscores the potential of low frequencies a‐tPCS, limitations regarding the effects of alpha (8–12 Hz), beta (15–36 Hz), and gamma (≥ 40 Hz) frequencies on CE and neural synchronization highlight the need for further research to deepen understanding and expand the clinical applications of tPCS.

### 4.5 Clinical Implications

The significant modulation of CE observed with low frequencies a‐tPCS suggests potential therapeutic applications for conditions characterized by abnormal cortical activity. This study indicates that a‐tPCS, as a neuromodulation technique, may have therapeutic effects on both hyperexcitability and degenerative disorders. The findings suggest that a‐tPCS at frequencies (≥ 1 Hz) may enhance CE and reduce intracortical inhibition, offering potential therapeutic benefits for individuals recovering from conditions such as stroke, depression, and other neurological disorders. Conversely, the inhibitory effects of a‐tPCS at frequencies (< 1 Hz) may be beneficial for conditions associated with cortical hyperexcitability, including anxiety, chronic pain, and epilepsy. These differential neurophysiological effects highlight the therapeutic potential of a‐tPCS, akin to TMS. However, the risk of seizure induction with TMS (Pascual‐Leone et al. 1998) limits its application in vulnerable populations, making tPCS a promising, safer alternative, particularly for at‐risk and diverse patient populations.

Compared to other non‐invasive neuromodulation techniques, tPCS appears to have unique advantages due to its frequency‐dependent effects, which likely involve both static and dynamic mechanisms. While tDCS and tACS also modulate CE through subthreshold mechanisms, they differ in their modulation patterns. TDCS predominantly induces static changes in CE, often producing more gradual and cumulative effects with less frequency dependence than tPCS or TMS. TACS, on the other hand, relies on dynamic mechanisms, as it entrains neural oscillations in a frequency‐dependent manner, making it particularly effective for modulating brain rhythms.

The potential for home‐based use of tPCS is promising, as it offers an affordable and accessible treatment option. However, several practical considerations must be addressed, including electrode placement accuracy, dose–response variability, and user compliance, all of which could impact real‐world effectiveness. Therefore, future research should not only evaluate the comparative safety and efficacy of tPCS relative to TMS, tDCS, and tACS but also investigate the feasibility of its clinical translation, particularly for home‐based applications.

## 5 Conclusions

This study provides evidence for the differential effects of low frequencies a‐tPCS on CE. The first aim aligns with TMS findings, which suggest that low‐frequency stimulation (< 1 Hz) is associated with LTD‐like effects, whereas higher frequencies (≥ 1 Hz) may facilitate LTP‐like mechanisms. However, it is significant to note that the observed changes in MEP amplitudes serve as indirect markers of synaptic plasticity and cannot definitively confirm LTD or LTP at a synaptic level.

Regarding the second aim, a‐tPCS at 1, 3, and 5 Hz significantly increased ICF, suggesting enhanced neuroplasticity, whereas 0.5 Hz heightened inhibitory neural activity by increasing SICI. These findings align with prior studies indicating frequency‐dependent modulation of excitability.

Finally, the third aim revealed that 0.5 Hz was associated with minimal adverse effects and an absence of phosphenes, while higher frequencies (1, 3, and 5 Hz) were associated with fewer side effects and phosphenes. Thus, future studies may consider incorporating phosphene perception as a potential method for individualizing tPCS intensity (Legros et al. 2024).

## Author Contributions

**Mona Malekahmad:** conceptualization, investigation, methodology, validation, visualization, software, formal analysis, project administration, data curation, resources, writing – review and editing, writing – original draft. **Ashlyn Frazer:** conceptualization, writing – review and editing, methodology. **Maryam Zoghi:** conceptualization, methodology, writing – review and editing. **Shapour Jaberzadeh:** conceptualization, supervision, formal analysis, writing – review and editing, methodology, visualization.

## Disclosure

*The use of generative AI and AI‐assisted technologies*: During the preparation of this work, the author(s) used ChatGPT in the writing process to improve the readability and language of the manuscript. After using this tool, the author(s) reviewed and edited the content as needed and take(s) full responsibility for the content of the published article.

## Conflicts of Interest

The authors declare no conflicts of interest.

## Data Availability

The data that support the findings of this study are available on request from the corresponding author. The data are not publicly available due to privacy or ethical restrictions.
